# Systematic review of gastrointestinal nematodes of horses from Australia

**DOI:** 10.1186/s13071-019-3445-4

**Published:** 2019-04-29

**Authors:** Muhammad A. Saeed, Ian Beveridge, Ghazanfar Abbas, Anne Beasley, Jenni Bauquier, Edwina Wilkes, Caroline Jacobson, Kris J. Hughes, Charles El-Hage, Ryan O’Handley, John Hurley, Lucy Cudmore, Peter Carrigan, Lisa Walter, Brett Tennent-Brown, Martin K. Nielsen, Abdul Jabbar

**Affiliations:** 1 Melbourne Veterinary School, The University of Melbourne, Werribee, VIC Australia; 2 School of Veterinary Science, University of Queensland, Gatton, QLD Australia; 3 School of Animal and Veterinary Sciences, Charles Sturt University, Wagga Wagga, NSW Australia; 4 School of Veterinary & Life Sciences, Murdoch University, Murdoch, WA Australia; 5 School of Animal and Veterinary Sciences, University of Adelaide, Roseworthy, SA Australia; 6 Swettenham Stud, Nagambie, VIC Australia; 7 Scone Equine Hospital, Scone, NSW Australia; 8 Boehringer Ingelheim Animal Health Australia Pty. Ltd, North Ryde, NSW Australia; 9 M.H. Gluck Equine Research Center, Department of Veterinary Science, University of Kentucky, Lexington, KY USA

**Keywords:** Gastrointestinal nematodes, Strongyles, Anthelmintic resistance, Horse, Australia

## Abstract

**Background:**

Equine gastrointestinal nematodes (GINs) have been the subject of intermittent studies in Australia over the past few decades. However, comprehensive information on the epidemiology of equine GINs, the efficacy of available anthelmintic drugs and the prevalence of anthelmintic resistance (AR) in Australasia is lacking. Herein, we have systematically reviewed existing knowledge on the horse GINs recorded in Australia, and main aspects of their pathogeneses, epidemiology, diagnoses, treatment and control.

**Methods:**

Six electronic databases were searched for publications on GINs of Australian horses that met our inclusion criteria for the systematic review. Subsets of publications were subjected to review epidemiology, diagnoses, pathogeneses, treatment and control of GINs of horses from Australia.

**Results:**

A total of 51 articles published between 1950 to 2018 were included. The main GINs reported in Australian horses were cyathostomins (at least 28 species), *Draschia megastoma*, *Habronema muscae*, *H. majus*, *Oxyuris equi*, *Parascaris equorum*, *Strongyloides westeri* and *Trichostrongylus axei* across different climatic regions of Queensland, New South Wales, Victoria, and Western Australia. Nematodes are diagnosed based on the traditional McMaster egg counting technique, though molecular markers to characterise common GINs of equines were characterised in 1990s. The use of anthelmintic drugs remains the most widely-used strategy for controlling equine GIN parasites in Australia; however, the threshold of faecal egg count that should trigger treatment in horses, remains controversial. Furthermore, anthelmintic resistance within GIN population of horses is becoming a common problem in Australia.

**Conclusions:**

Although GINs infecting Australian horses have been the subject of occasional studies over the past few decades, the effective control of GIN infections is hampered by a generalised lack of knowledge in various disciplines of equine parasitology. Therefore, coordinated and focused research is required to fill our knowledge gaps in these areas to maximise equine health and minimise economic losses associated with the parasitic infections in Australia.

**Electronic supplementary material:**

The online version of this article (10.1186/s13071-019-3445-4) contains supplementary material, which is available to authorized users.

## Background

Gastrointestinal nematodes (GINs) occur ubiquitously in horses (*Equus caballus*) and present a major veterinary concern throughout the world including in Australia. Strongylid (family Strongylidae) nematodes such as strongylins (large strongyles) and cyathostomins (small strongyles) are the main internal nematode parasites of horses constituting more than 75% of the total parasite fauna [1, 2]. Non-strongylid GINs found in horses include *Parascaris equorum*, *Habronema* spp., *Draschia megastoma*, *Oxyuris equi*, *Trichostrongylus axei* and *Strongyloides westeri* [3–5].

Due to conducive climatic conditions, GINs have been widely reported in horses from different agroclimatic regions across Australia [3, 4, 6–8]. These parasites have been associated with a variety of clinical signs, including unthriftiness, reduced stamina, retarded growth, abdominal distension (‘pot-belly’), diarrhea, abdominal pain and death, especially in young and immunocompromised horses [9–11]. Encysted cyathostomins cause larval cyathostominosis, a condition characterised by synchronous emergence of parasitic larvae from the large intestinal mucosa that can result in acute or chronic diarrhea, weight loss and, in some cases, death [4, 12–14].

Only limited information is available on the epidemiology and clinicopathology of equine GINs in Australia and the available information comes from selected regions. There are no detailed studies on equine GINs of horses in all different regions of the country which vary dramatically in climatic conditions from the northern tropical parts to the temperate southern areas. The impact of epidemiological factors on equine GIN prevalence and their clinical significance in Australia cannot be predicted based on the information from other regions of the world because of inter-continental climatic differences. Furthermore, climatic differences among regions within Australia might also have an effect on parasite epidemiology. GIN control in horses is traditionally based on regular anthelmintic administration to horses at intervals determined by egg reappearance periods which are likely affected by climatic conditions [15–18]. However, the majority of equine owners/managers in Australia usually follow a regular treatment interval of 6–8 weeks without any estimation of faecal egg counts (FECs) or consideration of seasonal variation [19]. The intensive use of anthelmintics has driven in the emergence of GIN populations resistant to all major classes of anthelmintics used in Australia, including benzimidazoles, macrocyclic lactones and tetrahydropyrimidines [12, 18, 20–24]. Thus, a national survey of the prevalence of anthelmintic resistance (AR) in Australian horses is required for the effective control and management of equine nematodes.

The aim of this article is to provide: (i) a systematic overview of the existing knowledge on the epidemiology, pathology and diagnosis of equine GINs in Australia; (ii) a thorough analysis of anthelmintic options and AR present in GINs infecting Australian horses; and (iii) areas for future research that could fill current knowledge gaps and enhance our understanding of equine GINs in Australia.

## Methods

The systematic review was conducted according to the Preferred Reporting Items for Systematic Reviews and Meta-Analyses (PRISMA) guidelines (see Additional file 1: Table S1). Inclusion and exclusion criteria were defined in terms of the relevance of the references to achieve the study objectives.

### Literature search

A systematic search was conducted utilising the Web of Science™ databases from 1950 to 2018 (accessed on December 18, 2018) to identify all publications reporting the GINs of Australian horses. Records of equine parasites prior to 1950 are summarised in the report of Mackerras [25]. Multiple key words were used to search for relevant articles, including endo-parasite*, endoparasite*, nematod*, gastrointestinal nematod*, strongyle*, cyathostomin*, anthelmintic*, anthelmintic resistance etc. and individual GIN names such as *Strongylus*, *Parascaris* etc. were also used. The search was refined by using primary (horse, equine) and secondary (Australia, Victoria, Queensland etc.) filters. The same keywords were used to search articles on other web browsers/databases, including PubMed, Google Scholar, Google, the library catalogue of the University of Melbourne and Australian online journal databases. Relevant articles were also identified from citations provided within the articles shortlisted in the primary search. A range of article types were considered for inclusion in this review, including as peer-reviewed original publications, conference papers, and post-graduate theses.

### Relevant screening, inclusion and exclusion criteria

**Fig. 1:**
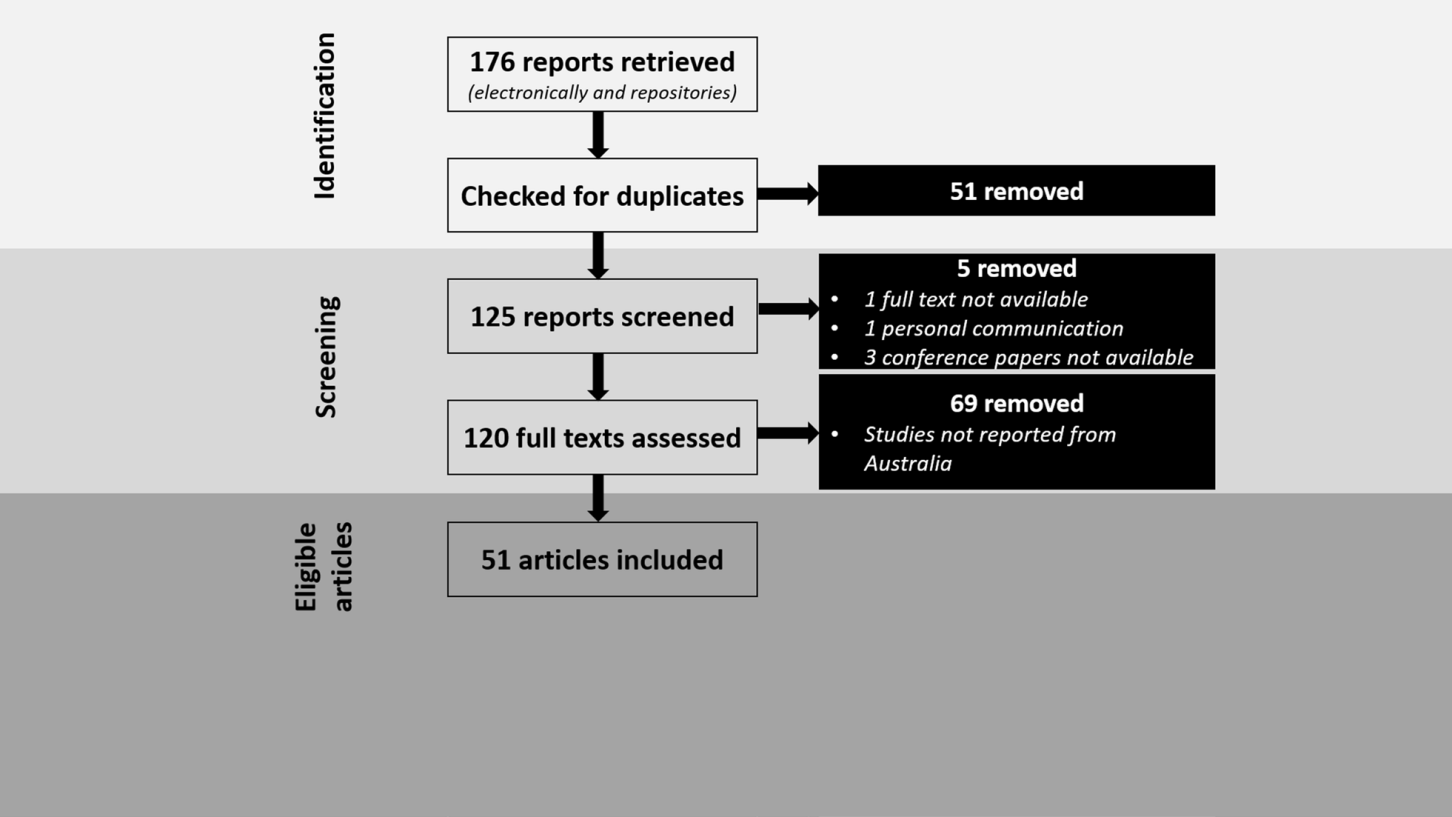
An overview of the assessment of peer-reviewed literature and the selection criteria used to select studies for this review paper

The literature assessment and selection criteria are illustrated in Fig. 1. In the primary assessment/selection based on the titles and abstracts, articles that aligned with the scope of this review article were shortlisted. Further screening and selection were performed to exclude those articles which were: (i) not full texts; (ii) personal communications without dates or publication details; and (iii) theses or conference articles not accessible in an online or published form. A total of 51 articles (all available in English) related to GINs, anthelmintic drugs or AR in horses from Australia were finally included in this review. All GIN parasites listed in this review article are sourced from the original reports/theses in Australia.

Registration numbers of voucher specimens of nematodes from Australian horses deposited in museum collections, both in Australia (The Queensland Museum, Brisbane, and The South Australian Museum, Adelaide) and in overseas collections (The United States National Parasite Collection, Smithsonian Museum, Washington), confirming identifications, are listed in the Additional file 2: Table S2.

## Strongylid nematodes

### Classification

The strongylids (family Strongylidae) are the most important parasites of adult horses and can be divided into two subfamilies based on their morphological characteristics [2, 26]. Species with globular or funnel-shaped buccal capsules are known as large strongyles or strongylins (subfamily Strongylinae), while species with cylindrical or ring-shaped buccal capsules are called small strongyles, cyathostomines, cyathostomins or cyathostomes (subfamily Cyathostominae) [2, 27]. Molecular phylogenetic studies to date, however, do not support this morphological division [28]. Adult strongylins are usually (but not always) larger in body size than the cyathostomins. Detailed keys for the identification of various genera of strongylid and non-strongylid nematodes of the horse have been provided elsewhere [2, 29, 30]. Multiple species occurring at a relatively high intensity of infection but dominated by positive interactions have been found in horses from Australia [1].

The Strongylinae comprises only 14 species grouped into five genera, including *Strongylus*, *Oesophagodontus*, *Triodontophorus*, *Bidentostomum* and *Craterostomum* [2, 26]. The Cyathostominae comprises more than 50 species grouped into 14 genera, including *Cyathostomum*, *Cylicocyclus*, *Cylicodontophorus*, *Cylicostephanus*, *Cylindropharynx*, *Gyalocephalus*, *Hsiungia*, *Parapoteriostomum*, *Petrovinema*, *Poteriostomum*, *Caballonema*, *Coronocyclus*, *Skrjabinodentus* and *Tridentoinfundibulum* [2, 26]. Importantly, in recent decades the cyathostomins have emerged as the most common nematode parasites and are important pathogens of adult horses worldwide [3, 9, 31, 32]. Overall, the cyathostomins share a similar morphology, epidemiology and disease biology [2]; thus, they have been discussed as a single entity for this review.

### Life-cycles

Like many GINs, strongylids undergo a direct life-cycle with adults residing in the caecum and colon of horses [9, 26]. Eggs laid by adult females are shed within the faeces and subsequently hatch to first-stage larvae (L1) in the environment under optimum conditions of temperature and humidity. Following two moults, larvae mature to infective third-stage larvae (L3) which are ingested by horses during grazing. After exsheathment within the small intestine, larvae mature through various stages (L4 and L5/adult) finally developing into adults within the caecum/colon of horses. However, following exsheathment, the larvae of *Strongylus* spp. undergo an extra-intestinal migration while those of cyathostomins, *Craterostomum*, *Triodontophorus* and *Oesophagodontus* burrow into the colonic and cecal mucosa and subsequently become encysted with no extra-intestinal migration [9, 26, 33].

### Epidemiology and predisposing factors

#### Prevalence and distribution

At least 28 species of cyathostomins have been reported across different climatic regions of Queensland, New South Wales, Victoria and Western Australia (Table 1). A complete list of GIN prevalence, infection intensity and method of detection in individual studies as well as identification numbers of equine parasites collected from Australia and deposited at various museums, have been provided in Additional file 2: Table S2.

**Table 1:** Gastrointestinal nematodes identified from horses in Australia

Parasite type/spp.	Location (s)	Prevalence range (%)	Reference
Cyathostomins			
*Coronocyclus* spp. (*Co. coronatus*, *Co. labiatus*, *Co. labratus*)	QLD, NSW, VIC, WA	4–76	[3, 4, 7, 8, 34, 69, 101]
Unidentified cyathostomins	QLD, WA	Up to 49	[5, 39]
*Cyathostomum* spp. (*Cy. catinatum*, *Cy. pateratum*)	QLD, NSW, VIC, WA	Up to 76	[3, 4, 8, 34, 43, 69, 70, 101]
*Cylicocyclus* spp. (*Cc. ashworthi*, *Cc. auriculatus*, *Cc. brevicapsulatus*, *Cc. elongatus*, *Cc. insignis*, *Cc. leptostomus*, *Cc. nassatus*, *Cc. radiatus*, *Cc. ultrajectinus*)	QLD, NSW, VIC, WA	1–72	[3, 4, 8, 34, 69–71, 101, 102]
*Cylicodontophorus bicoronatus*	QLD, NSW, VIC	3–4	[4, 8, 34, 101, 102]
*Cylicostephanus asymmetricus*, *Cs. calicatus*, *Cs. goldi*, *Cs. hybridus*, *Cs. longibursatus*, *Cs. minutus*	QLD, NSW, VIC, WA	2–76	[3, 4, 8, 28, 34, 69, 101, 102]
*Gyalocephalus capitatus*	QLD, VIC	1–11	[4, 8, 34, 40]
*Parapoteriostomum* spp. (*Pp. euproctus*, *Pp. mettami*)	QLD, NSW, VIC	1–15	[4, 8, 34, 101, 102]
*Petrovinema poculatum*	QLD, VIC	2–9	[4, 8, 34, 102]
*Poteriostomum* spp. (*P. imparidentatum*, *P. ratzii*, *P. skrjabini*)	QLD, NSW, VIC	1–52	[4, 8, 34, 69, 101]
“Strongyles”	QLD, VIC	Up to 52	[11, 43]
Strongylins			
*Craterostomum acuticaudatum*	VIC	7	[4]
*Oesophagodontus robustus*	QLD, VIC	Up to 2	[8, 34, 69, 102]
*Strongylus* spp. (*S. edentatus*, *Strongylus equinus*, *Strongylus vulgaris*)	QLD, VIC, WA	3–88	[4, 5, 8, 29, 34, 36, 37, 40, 69]
*Triodontophorus* spp. (*T. brevicauda*, *T. minor*, *T. nipponicus*, *T. tenuicollis*, *T. serratus*)	QLD, VIC, WA	3–30	[3, 4, 8, 34, 37, 40, 69, 102]
Non-strongylids			
*Draschia megastoma*	QLD, VIC, QA	5–66	[4, 5, 7, 51, 55]
*Habronema* spp. (*H. muscae*, *H. majus*)	QLD, VIC, QA	2–72	[3–5, 7, 51]
*Trichostrongylus axei*	VIC	51	[4]
*Oxyuris equi*	QLD, VIC, QA	7–26	[3–5, 7]
*Parascaris equorum*	NSW, QLD, VIC, QA	5–58	[3–5, 7, 20]
*Strongyloides westeri*	QLD, VIC	6	[7, 29]
*Probstmayria vivipara*	QLD	2	[7]

*Abbreviations*: EPG, eggs per gram feces; na, not available/applicable; NSW, New South Wales; SA, South Australia; VIC, Victoria; WA, Western Australia

Cyathostomins are present in the majority (˃ 70%) of horses surveyed with multiple species typically present in individual horses [4, 34]. Some of these species (e.g. *Cyathostomum catinatum*, *Cylicocyclus nassatus* and *Cylicostephanus calicatus*) have been found more frequently (prevalence ˃ 70%) in horses from Australia compared with the other species [3, 34]. Likewise, variable infection levels for individual cyathostomin species have been observed, ranging from a few to more than 100,000 worms in individual horses (see Additional file 2: Table S2).

Although adult strongyles reside in the caecum and colon, the infection intensity and distribution of various species can vary between different parts of the large intestine and is indicative of the site preference. For instance, the majority of worms have been found in the ventral colon, followed by the dorsal colon and the caecum in Australian horses [34]. *Cylicostephanus poculatus* preferentially inhabits the caecum while *Coronocyclus labratus*, *Cylicocyclus insignis*, *Cylicostephanus asymetricus* and *Oesophagodontus robustus* are found in the ventral colon and *Cylicostephanus hybridus*, *Parapoteriostomum mettami* and *Posteriostomum skrjabini* occur most often in the dorsal colon of horses [34]. Similarly, *Cs. longibursatus*, *Cs. goldi*, and *Cs. minutus* are found more frequently in the large colon (i.e. dorsal and ventral colon) and less often in the caecum and small colon [4]. Although differences may exist in the distribution of some species (e.g. *Co. labratus*) in horses from Australia [4] compared to the UK [35], no associations have been found between the variations in site preference and the age, sex or breed of horse or the environment (i.e. wet *vs* dry tropics, coastal *vs* subcoastal, paddocked *vs* stabled horses) [34]. However, seasonal variations may affect the site distribution and there could be an increase in the proportion of parasites in regions anterior or posterior to the preferred site during a particular season [34]. For instance, some strongyle parasites which typically prefer the dorsal colon appear with increased frequency in the caecum and/or ventral colon of horses during summer and autumn in tropical Australia [34].

Likewise, *Coronocyclus coronatus* (which generally prefers to reside in the caecum) is more commonly found in the ventral colon in summer and winter in tropical Australia but shifts to the dorsal colon in autumn and spring [34]. These authors hypothesised that the site preference of cyathostomin species could also be related to the diet of the parasite and stage of parasite development. For instance, strongylins are usually tissue feeders and the cyathostomins feed on the ciliate and bacterial populations in the large intestines of horses [34]. A thorough understanding of site distribution of preferences may play a crucial role in elucidating the overall pathogenesis of disease, but it should be noted that these differences could be a result of differences in examination methods.

#### Effect of environmental factors

According to the current epidemiological information of GINs of sheep in Australia, and the temperature and rainfall in different parts of the country, climatic zones can be categorized into eight zones, including (i) Western Australian winter rainfall; (ii) South Australian winter rainfall; (iii) Victorian winter rainfall; (iv) Tasmania; (v) New South Wales non-seasonal rainfall; (vi) Queensland/New South Wales summer rainfall/slopes and plains; (vii) New South Wales/Queensland summer rainfall/tablelands and slopes; and (viii) Pastoral (http://www.wormboss.com.au). The Australian climate can be divided into summer (December–February), autumn (March–May), winter (June–August) and spring (September–November).

For GINs of horses, faecal pats act as reservoirs for nematode larvae and play a crucial role in herbage contamination which is seasonal and influenced by moisture content [10, 36, 37]. For example, in dry tropical and subtropical areas of Queensland, strongyle larvae survive longer under cool conditions (i.e. winter) than during hot summers (wet) with the presence of adequate moisture to keep faecal masses moist [10, 38]. Higher survival rates (over 80%) have consequently been observed in winter compared with summer (1–10%), although larvae may take longer (e.g. 5 weeks) to moult to the L3 in winter compared to only one week in summer [10]. This suggests that short-term pasture spelling may not be adequate to control strongyle nematodes in horses in these regions, particularly in winter and autumn. Larvae accumulate gradually on pasture and can reach up to 60,000 larvae kg^−1^ under optimum climatic conditions [38]. Heat and moisture promote larval migration to herbage and horses in the dry tropics of Queensland are at higher risk of acquiring an infection during and immediately after the wet season [38]. Treatment, therefore, during hot weather immediately prior to the onset of wet season could reduce pasture infectivity later [38, 39]. Furthermore, unseasonable rainfall and the presence of swamps or irrigation areas may increase the infectivity of the pasture during a dry season [38].

The number of larvae reaching the infective stage (i.e. L3) and the length of their survival/viability in faeces are important epidemiological parameters in determining the acquisition of strongyle infections in horses. For instance, in horses from tropical Queensland, peak FECs have been observed during the late summer through autumn with a slight decline in winter with the lowest FECs recorded in spring to early summer [39]. It is important to consider that seasonal variation could also affect the proportions of different strongyle species in horse faeces. For instance, a peak and marked reduction in FECs for cyathostomins and *Strongylus* spp., respectively, have been observed in late summer through autumn periods [39]. Likewise, horses from wet coastal and inland areas carried more strongyle species per horse and higher worm burdens compared with those from dry coastal areas [8].

In Victoria, the highest prevalence of cyathostomins has been observed in summer, followed by winter and autumn, although the highest worm burdens were noted in autumn [4]. Furthermore, variations were observed in the prevalences of individual parasite species in different seasons. For instance, *Cy. pateratum* and *Cc. leptostomus* had higher prevalences in autumn than in summer or winter while *Cy. pateratum* was less prevalent during autumn. Likewise, *Cd. bicoronatus*, *Cy. pateratum*, *Co. labiatus*, *Cs. calicatus* and *Cc. insignis* were more prevalent in areas of uniform high rainfall compared to areas with low-to-medium winter rainfall [4]. *Cyathostomum catinatum*, *Cy. pateratum* and *Cc. leptostomus* had higher mean worm burden in horses from areas of medium rainfall [4].

The availability of strongylid nematode larvae on pasture over a 12-month period has been examined at Werribee, Victoria [40]. Peak larval (4500 larvae/g of herbage) numbers occurred in autumn, with low numbers in winter and spring and a gradual increase in numbers in late summer. Faecal egg output in horses was also followed and recorded a mean maximum of 3100 EPG (eggs per gram of faeces) again in autumn, a decline in winter with a subsequent rise again the following spring [40]. It should be noted that the horses in this study received a single anthelmintic treatment in winter when egg counts were already low.

In tropical Queensland, increased infection intensities have been observed for *S. vulgaris* during mid-winter through late spring and *S. edentatus*/*S. equinus* during late spring through summer [39]. *Strongylus equinus* is extremely rarely reported these days. In Victoria, *S. vulgaris*, *S. edentatus* and *T. serratus* exhibited some seasonal variations in prevalence but no variations in the mean intensity of infection. For instance, *S. vulgaris*, *S. edentatus* and *Triodontophorus* spp. were more prevalent in areas of uniform high rainfall compared to areas of winter low/medium rainfall [4]. Knowledge of the impact of seasonal variations on pasture contamination (i.e. larval ecology) and parasite prevalence may assist in the development of rational anthelmintic control programmes. However, it is clear that this will require detailed knowledge of the seasonal variations in both the prevalence and intensity of parasitic infections across a range of climatic zones.

#### Host factors

Host or biotic factors such as age, sex and physiological/immunological status of the horse can play an important role in the acquisition and pathogenicity of GIN infections [9, 41, 42]. However, there is a paucity of information on the influence sex, breed or age on the infection by strongylid parasites in horses from Australia and elsewhere.

In Victoria, the highest infection intensity of cyathostomins has been observed in geldings, although they exhibited a lower parasite prevalence than male or female horses [4]. For instance, the highest infection intensity of *Cylicostephanus* spp. and *Cyathostomum* spp. were observed in geldings followed by female and male horses. However, *Cs. calicatus* exhibited a higher prevalence in males in contrast to *Cs. goldi* which was more common in females [4]. Similarly, *T. serratus* or *T. tenuicollis* were observed only in male and female horses with no infections detected in geldings [4]. Conversely, no associations have been documented between the sex of the horse and the number of strongyle eggs in faeces in horses from Victoria [43]. Further studies are obviously required to investigate the precise effect of sex on the prevalence and intensity of strongyle nematode infections.

Age is also important in determining GIN prevalence in animals including horses. Younger horses (˂ 2 years) have been found to acquire *Co. coronatus*, *Co. labratus*, *Cs. calicatus*, *Cs. poculatus* and *Cc. elongatus* infections more frequently than older horses [4]. Conversely, *Co. labiatus* and *Cc. insignis* were more common in horses between 2–7 and 7–15 years of age respectively. *Cyathostomum* spp. showed an increasing worm burden with the age, peaking in horses aged between 2–7 years and then decreasing in older horses [5]. Conversely, no associations have been noted between the age of horse, the prevalence of cyathostomins and strongylins, including *S. vulgaris* and *S. edentatus* [5, 8]. Other studies have shown a higher prevalence of *T. serratus* and *T. brevicauda* in young horses (˂ 2 years) and *S. edentatus* in horses between 2–7 years of age from Victoria [4]. Overall, these data warrant further investigation into the effect of age on strongyloid prevalence and abundance in Australia.

No significant correlations have been found between horse breed (e.g. Standardbreds, Thoroughbreds, Quarter Horses) and prevalence of cyathostomins [8]. However, Thoroughbred horses generally harbored larger worm burdens than the other types of horses [8]. Likewise, *Triodontophorus* spp. have been observed more frequently in Thoroughbred compared to Standardbred horses, although the reverse is true for *S. equinus* [4]. It should noted that none of the cited studies were designed to evaluate the effect of horse breed on equine parasitism, so these results should be interpreted with great caution.

### Pathogenesis

It is difficult to dissect the role of individual cyathostomin species given that they occur in naturally acquired mixed infections on associated pathogenesis in horses [4, 5, 43]. However, in large numbers, adult cyathostomins appear to induce intestinal pathology in at least some infected horses and infection has been associated with weight loss, chronic colic, lethargy, debilitation and diarrhea [44, 45]. Larval stages are pathogenic due to invasion of the intestinal wall, resulting in inflammation and damage to the mucosa [44]. A wall protects encysted larvae and their emergence from cysts in large numbers especially in the late winter or early spring can cause severe damage and the associated clinical syndrome is termed “larval cyathostominosis”. This condition is characterised by pyrexia, edema, protein loss, diarrhea, weight loss and death [9, 46]. Most cyathostomin infections in horses from Australia have been reported as asymptomatic [4, 34, 43] and larval cyathostominosis is not very common in Australia as well as in the rest of the world.

Adult strongylins are considered pathogenic since they attach to the host’s intestinal wall (for blood-feeding) which can result in mechanical rupture of the tissue and blood vessels. Most *Strongylus* spp. and *Triodontophorus* spp. observed in post-mortem samples from horses in Queensland were firmly attached to the intestinal mucosa in contrast to freely residing (between the intestinal contents and the mucosal lining) cyathostomins [34]. The larval stages of *S. edentatus* and *S. equinus* can induce physical injury and inflammation in organs, including the liver, pancreas and peritoneum during migration. This has been associated with a range of clinical signs such as inappetence, pyrexia and colic [47, 48]. Larval migration of *S. vulgaris* through arteries causes ‘verminous arteritis’ characterised by a pronounced influx of inflammatory cells into vessel walls with subsequent intimal thickening [48]. Heavy burdens of *S. vulgaris* have been associated with severe mesenteric arteritis in horses from various regions of Australia [5, 29, 37, 49]. Thrombi (and emboli) within mesenteric arteries can obstruct blood flow resulting in infarction of intestinal walls and colic [48]. Increased colonic and caecal motility have also been observed in *S. vulgaris* infections in horses [50].

Studies on strongylins from Australian horses indicate that most species exhibit some degree of site preference. For instance, *S. vulgaris* and *S. edentatus* reside more frequently in the caecum/ventral colon and large/dorsal colon, respectively in horses from Victoria [4] and Queensland [34]. Similarly, *S. equinus* and *Oesophagodontus robustus* have been predominantly found in the ventral colon of horses from Queensland [34]. No meaningful correlations have been established between the severity of the arteritis or larval burden within the arteries and signalment or season in horses from Australia [5, 37].

## Non-strongylid parasites

### Stomach worms

The four species of nematodes that can be found in the stomach of horses are *Draschia megastoma*, *Habronema muscae*, *H. majus* and *Trichostrongylus axei* [3, 5, 51, 52]. *Draschia megastoma* and *Habronema* spp. share similar morphological characteristics and belong to the superfamily Spiruroidea. Unlike strongyles, the spirurid nematodes exhibit an indirect life-cycle with the requirement of an intermediate host, i.e. *Musca domestica* (housefly) or *Stomoxys calcitrans* (stable fly). In Western Australia, a peak in spirurid larval prevalence has been observed in house flies in spring and early summer but no larvae were detected in stable flies over the same period [5]. Spirurid eggs containing the L1 larvae are passed in horse faeces subsequently ingested by maggots and develop synchronously with the fly larvae to reach the infective L3 stage in about two weeks. Infected flies deposit L3 larvae around the horse’s mouth which are then swallowed by the horse and develop to adult worms in the stomach over two months [52–54].

*Draschia megastoma* is the most pathogenic of the three spirurid species and has been associated with multiple nodular granulomas (1–7 cm) in the stomach of horses from Western Australia [5]. Submucosal eosinophilic granulomas are usually located near the *margo plicatus* and eventually coalesce to form large fibrous masses [5, 52]. In most cases, small nodules have no effect on the stomach motility; however, large nodules can interfere with gastric function and have been suggested to cause stomach rupture [5, 52]. *Draschia megastoma* infections have been associated with splenic abscesses and adhesions between the spleen and stomach of horses in New South Wales and Western Australia [55]. A significantly higher prevalence of *D. megastoma* lesions has been observed from late winter through early summer with a second peak in autumn in horses from Western Australia [5]. However, no association could be established between the prevalence of adult *D. megastoma* and the age of horses [5]. In horses from Victoria, the highest infection intensities of adult and larval members of the Habronematidae have been observed in autumn, followed by winter and summer. Furthermore, males had a higher infection intensity for habronematid larvae than females or geldings [4].

Habronemosis is an important parasitic disease of equids including horses caused by the larvae of *Habronema* spp. [53]. *Habronema* spp. are known to cause pathologies in horses especially of the stomach wall where they can stimulate copious, thick glandular secretions particularly from areas closer to the *margo plicatus* [52, 53]. However, mostly *Habronema* infections reported in Australian horses are asymptomatic with no significant pathologies identified [3–5, 7, 51]. Third-stage larvae of both *D. megastoma* and *Habronema* spp. can infect the skin and the region around the eye causing ‘summer sores’ or cutaneous habronemiasis [55].

Subclinical infections of *T. axei* have been observed in horses from Victoria. *Trichostrongylus axei* was more prevalent in horses older than two years of age and in areas with low-to-medium winter rainfall [4]. *Trichostrongylus axei* is one of the economically important GINs of domestic livestock and it can be an important parasite of horses where co-grazing occurs.

### Ascarids (roundworms)

*Parascaris equorum* and *P. univalens* are important equine roundworms belonging to the family Ascarididae, the members of which are commonly known as ascarids [56–58]. *Parascaris equorum* is a large parasite that measures up to 28 cm in length for male worms and 50 cm for females with three large lips. The parasite is found in the small intestine of horses and undergo a direct life-cycle [29]. Development to the infective L3 larva occurs within an egg and can be completed in 9–14 days under optimum conditions (i.e. 25–35 °C) but is arrested if temperatures fall below 10 °C. Importantly, eggs can survive for years on pasture unless exposed to high temperatures (above 39 °C) [56, 59]. Following ingestion, embryonated eggs hatch in the horse’s small intestine, penetrate the intestinal mucosa and migrate to the liver and lungs. Larvae then migrate up to the respiratory tree to the pharynx, are swallowed, and finally develop to mature adult worms within the duodenum and jejunum. The prepatent period is between 75–115 days [56, 57, 59].

Clinical signs associated with *P. equorum* infections include lethargy, inappetence, reduced weight gain, coughing, nasal discharge, oedema and colic in young horses [59]. Ascarid impactions of the small intestine are a well-recognised phenomenon often requiring intestinal surgery and typically carrying a guarded prognosis [59]. Necrotising enteritis, septic peritonitis and intestinal rupture can also be seen on necropsy of parasitized horses [57, 59]. *Parascaris equorum* infections have been reported in horses from New South Wales [20], Queensland (especially coastal areas) [7], Victoria [4] and Western Australia [3, 5] with a prevalence as high as 58%. Infections are significantly more common in horses under two years of age [4, 5, 7]. Higher prevalences are observed during summer, in paddocked rather than stabled horses [7], and in males compared to gelding and female horses [4]. Interestingly, despite their pathogenicity, *P. equorum* infections in Queensland and Western Australia were not associated with any gross pathological changes [5, 7].

### Oxyurids (pinworms)

*Oxyuris equi* is a common pinworm with a worldwide distribution in horses [3, 60, 61]. Like many other nematode parasites, *O. equi* undergoes a direct life-cycle with adults residing mainly in the dorsal colon and adjoining regions with heavy infections. Eggs are deposited within an adhesive medium onto the perianal region which are visible grossly as yellowish white gelatinous streaks on the skin. These can cause irritation and pruritus which consequently leads to rubbing and disruption of the tail hair. Feeding of the larval stages within the mucosal crypts of the intestine of horses can lead to erosions and an influx of inflammatory cells into the mucosa [61].

In Australia, the fourth-stage larvae of *O. equi* have been recovered more frequently than adult worms in horses from Queensland [7], Victoria [4] and Western Australia [3, 5]. Furthermore, high infection intensities (over 10,000 larval worms per horse) have been observed in horses from Australia [3]. Since the prepatent period of this parasite is about five months, the scarcity of adult *O. equi* in these studies may reflect the regular use of anthelmintics which may inhibit development of adults from larvae [3]. Additionally, the development of *O. equi* appears to be favored by high rainfall, as higher infection rates with adult worms have been observed in horses from areas with uniform high rainfall [4, 7].

The small pinworm, *Probstmayria vivipara*, of horses has also been reported when Mfitilodze & Hutchinson [7] undertook necropsies of 57 horses from Queensland (see Additional file 2: Table S2).

### Threadworms

*Strongyloides westeri* (family Strongyloididae) is a threadworm parasite found in young foals up to approximately 16 weeks of age [62]. Foals can acquire infection from their dam’s milk or through percutaneous invasion and migration via the lungs. It should be noted that migration through the lungs rarely causes overt respiratory illness [62]. *Strongyloides westeri* undergoes a direct life-cycle with a prepatent period of 8–15 days. Adult parasites inhabit the small intestine where they (only females) can induce enteritis, characterised by erosions, catarrhal lesions and edema of the mucosa that may result in impaired digestion, malabsorption and diarrhea [58, 62].

A low prevalence (6%) of *S. westeri* has been observed in foals from Queensland [7]. The inclusion of limited number of very young foals in this study could be a potential reason for the reported low prevalence as most foals become resistant to the infection by 16 weeks of age [7, 62]. However, the parasite might be more widespread that recognized as it is anecdotally regarded as a common parasite in horses from some regions of the country [29].

## Diagnosis

Accurate diagnosis of GIN infections of horses is crucial in parasite surveillance and control programmes. Suspicion of a GIN infection might be based on clinical signs (e.g. diarrhea, colic), age, inappropriate anthelmintic administration (either inadequate or excessive administration) and the presence of reproductive stages of parasites in the faeces. The detection of *Oxyuris* eggs in a smear from the perianal region of a horse with a history of tail rubbing is generally considered sufficient to confirm the diagnosis of oxyurosis [60]. The diagnosis of strongyloid infections in live horses is usually made by simple faecal floatation test, using various saturated floatation solutions. However, the clinical significance of infection is often difficult to determine and the absence of parasitic eggs within the faeces does not exclude parasitic disease [33]. A flotation solution such as sucrose, sodium nitrate, sodium sulfate, sodium chloride, zinc sulfate, or magnesium sulfate provides a suitable specific gravity that helps to separate parasite eggs from faecal debris. Rather than single faecal samples, wider faecal sampling (both spatially and temporally) likely allows a better estimation of the distribution pattern of egg shedding but this is obviously difficult when evaluating an individual horse with suspected parasitic disease [63]. Various tests are used to quantify the number of parasite eggs (eggs per gram of faeces: EPG) in horse faeces and provide an estimate of parasitic burden. These quantitative tests include the McMaster, FECPAK, FLOTAC and the Ovatec methods. However, they differ in precision and accuracy, and the requirement for specialised laboratory equipment or technical expertise to perform the test [16]. Novel smartphone-based automated parasite faecal egg counting techniques appear to provide accurate, precise and rapid quantification of strongyle eggs in horse faeces and could be useful in monitoring egg shedding in equines [64, 65].

Studies have demonstrated low correlation between FECs and the number of cyathostomins present within the lumen of horse’s large intestine. For example, horses can carry populations of encysted larvae without any detectable luminal worms and, because immature worms do not produce eggs, FECs can be negative [3]. Conversely, egg counts can be misleading in the case of *P. equorum* infections due to extreme fecundity of the mature worms [59]. Therefore, the results of faecal examinations should be interpreted carefully as egg counts are not useful as clinical diagnostic tools because the pathogenic stages generally are larvae, not egg producing adults. Furthermore, there is no correlation with the adult worm burden and FECs [9, 66, 67]. The differentiation of strongyle eggs based on morphological identification is not possible but larval culture allows the identification of third-stage larvae (e.g. *S. vulgaris*) that can provide more detailed information about the prevalent parasitic species/genus [67]. However, it can take 1–2 weeks to accomplish for equine strongyles. Contrarily, molecular approaches offer a reliable alternative to morphological identification of GINs in horses as they are more accurate and rapid [33].

A molecular test has been developed to differentiate eggs of *Strongylus* spp. in horse faeces [68]. This test is based on detecting interspecific differences in the internal transcribed spacer (ITS)-2 sequences of *Strongylus* spp. which allows the identification of individual species from a single worm or egg. The test utilizes polymerase chain reaction (PCR) methodology in conjunction with restriction fragment length polymorphism (RFLP) analysis. Similar approaches have been used for identification and phylogenetic analyses of a range of strongylin and cyathostomin parasites as well as differentiation of closely related cyathostomin species [28, 33, 69–73]. Species-specific amplification of parasitic ribosomal DNA has been performed successfully from horse faeces [70]. A mutation scanning approach based on single-strand conformation polymorphism (SSCP) has been used to identify ascarid parasites including *P. equorum* [74]. These studies suggest that PCR-based methods targeting equine ITS gene sequences could be highly valuable for diagnosis, species identification, and phylogenetic analyses of equine strongyle parasites.

## Control of GINs in horses

### Anthelmintic treatment

The three major anthelmintic classes used for GIN control in Australian horses are benzimidazoles (e.g. fenbendazole and oxfendazole); macrocyclic lactones (MLs; e.g. abamectin, ivermectin and moxidectin); and tetrahydropyrimidines (e.g. morantel and pyrantel). Drugs within each of these classes have variable levels of anthelmintic activity, efficacy, and duration of activity (Table 2).

**Table 2:** Anthelmintics used against gastrointestinal nematodes of horses in Australia

Anthelmintic agent	Dosage (mg/kg)^a^	Efficacy (FECR %)	Days post-treatment	Parasite	Reference
Abamectin + praziquantel	0.2, 2.3	≥ 90	28	Strongyles	[15]
Ivermectin	0.2	≥ 90	42	Cyathostomins	[78]
	0.2	≥ 90	42	Cyathostomins, *Gyalocephalus* spp., *Triodontophorus* spp.	[17]
	0.2	≥ 90	42	Cyathostomins	[23]
	0.2	na	42	Strongyles	[75]
Ivermectin + praziquantel	0.2, 1.5	≥ 90	42	Cyathostomins, *Gyalocephalus* spp., *Triodontophorus* spp.	[17]
Moxidectin	0.4	≥ 90	84	Cyathostomins	[78]
	0.4	≥ 90	42	Cyathostomins, *Gyalocephalus* spp., *Triodontophorus* spp.	[17]
	0.4	≥ 90	84	Cyathostomins	[23]
Moxidectin + praziquantel	0.4, 2.5	≥ 90	84	Strongyles	[15]
Fenbendazole	10	100	49	Strongylins	[18]
Oxibendazole	10	≥ 90	14	Cyathostomins	[23]
	10	100	49	Strongylins	[18]
Thiabendazole + piperazine + trichlorphon	44, 125, 40	100	20	Cyathostomins	[80]
Morantel	9.4	≥ 90	14	Cyathostomins	[23]
	10	≥ 90	27	Cyathostomins	[18]
	10	100	49	Strongylins	[18]
	12.5	99	20	Cyathostomins	[80]
	10	100	7	Cyathostomins	[79]
Morantel + abamectin	9, 0.2	100	56	*Parascaris equorum*	[24]
Morantel + oxibendazole + dichlorvos	10 each	99	27	Cyathostomins	[18]
	10 each	100	49	*P. equorum*, Strongylins	[18]

^a^Oral route

*Abbreviations*: FECR, fecal egg count reduction; GINs, gastrointestinal nematodes; na, not applicable/available

Oral formulations of ivermectin (0.2 mg/kg) have been found effective in reducing strongyle (cyathostomins, *Gyalocephalus* spp., *Triodontophorus* spp.) burdens to below 90% for up to 6 weeks post-administration [17, 23, 75, 76]. Praziquantel is included in some anthelmintic formulations for control of cestode parasites. Although ivermectin exhibits a broad spectrum of activity against various luminal parasites including adult and larval stages of GINs, encysted cyathostomin larvae are refractory to ivermectin treatment. Even at higher drug concentrations, ivermectin has been demonstrated to be ineffective at treating encysted larval cyathostomins [23]. Thus, ivermectin is unlikely to successfully treat encysted cyathostomin larvae, even at higher drug concentrations [9, 77]. Conversely, moxidectin (0.4 mg/kg) currently has efficacy against all stages of cyathostomins and thus limits reinfection with these parasites. For instance, moxidectin (0.4 mg/kg; oral) gel has been found effective to reduce strongyle eggs to below 100 EPG (FECR ≥ 90%) for at least 12 weeks following administration, despite continuous reinfection in horses from the pasture [15]. Furthermore, moxidectin was found effective in reducing strongyle (cyathostomins, *Gyalocephalus* spp., *Triodontophorus* spp.) burdens below 90% for as long as 12 weeks post-administration [17, 23, 78]. The longer anthelmintic treatment interval required for moxidectin could be highly valuable in reducing treatment frequency, and consequently selection of AR [9]. However, consideration needs to also be given to the longer duration of therapeutic levels of moxidectin which could increase the risk of selecting for AR, especially in low refugia situations.

Other MLs such as abamectin have been used in combination with praziquantel alone or in combination with oxfendazole or morantel, with high efficacy against stronglylins, cyathostomins and *Parascaris* spp. in horses from Australia. However, resistance of *Parascaris* spp. has been reported to an abamectin/morantel combination where abamectin alone exhibited poor efficacy [18, 24, 79, 80]. A combination of morantel, oxibendazole and dichlorvos was shown to be effective against cyathostomins, strongylins as well as a benzimidazole-resistant *P. equorum* [18]. Dichlorvos is no longer available as a commercial paste due to occupational health and safety issues.

The use of anthelmintic drugs remains the most widely-used strategy for controlling equine GIN parasites in Australia and elsewhere [15, 17–19], however, the threshold FEC that should trigger treatment in horses, remains controversial. Suggested threshold values in the literature vary and include 100 EPG [15, 81], 200 EPG [17, 82], between 200–500 EPG [16], and 10% of the pre-treatment EPG values [83, 84]. Furthermore, the mean efficacy < 95% during the first 2–3 weeks post-treatment in horses has been regarded as an indicator of reduced efficacy of the anthelmintic used [84]. Therefore, an agreeable definition for ERP and cut-off FECRT values in horses in Australia and worldwide is needed.

### Non-anthelmintic control methods used in Australia

Although there are limited studies reported in the peer-reviewed literature, non-anthelmintic options have been trialed for the control of equine GINs in Australia. The use of nematophagous fungi represents one novel approach to reduce free-living larval stages of animal parasites including those of horses. The chlamydospores of the fungus *Duddingtonia flagrans*, added to animal feed, pass through the digestive tract and inoculate the faeces. Once passed, the fungus germinates, and fungal hyphae form a network (mesh) of sticky traps throughout the faecal mass which inhibits the development of free-living larval stages reducing the incidence of infection in grazing animals. The effectiveness of chlamydospores (3 × 10^4^ spores/kg) of *D. flagrans* strain IAH 1297 has recently been trialed in horses from New South Wales [85]. This resulted in a 53–94% reduction in parasite larvae (principally cyathostomins, *Strongylus* spp. and *T. axei*) on the pasture over the eight weeks of the study across several different climatic conditions [85]. Given that this type of approach could be a valuable alternative in GIN control, further studies are urged to evaluate the effect of nematophagous fungi on equine GINs in additional agroclimatic regions of Australia.

Crude extracts from Australian native plants such as *Acacia baileyana*, *Acacia melanoxylon*, *Acacia podalyriifolia*, *Alectryon oleifolius*, *Duboisia hopwoodii*, *Eucalyptus gomphocephala* and *Santalum spicatum* have been shown to be highly effective (up to 100%) in inhibiting larval (cyathostomins) development *in vitro* [86]. Subsequent studies have demonstrated that procyanidin A2 is the active compound in *A. oleifolius* plant extracts [87]. Procyanidin exhibits significant anthelmintic activity and inhibited larval development and larval migration at concentrations as low as 50 µg/ml and 25 µg/ml, respectively [87]. These studies highlight the potential for the integration of these plants into equine parasite control programmes, although field trials in horses are required.

Pastures management could play a key role in the transmission of nematode parasites of horses in Australia. For example, horses from higher rainfall areas and those that graze pasture year-round are usually exposed to higher levels of infective larvae than those from drier areas or horses that are frequently stabled. Furthermore, horses at pasture and those in higher rainfall areas exhibit higher prevalence rates, infection intensities, and harbour a broader range of GIN species [10, 14, 37]. Thus, preventing the deposition of larvae on pasture during climatically favorable periods of the year could significantly help to reduce GIN burdens in horses. Susceptible horses should also be removed from heavily contaminated pastures during the periods when high pasture larval burdens are anticipated. For instance, in tropical/subtropical areas of Queensland, the risk of GIN infections in horses could be reduced if pasture contamination is prevented or limited in spring and autumn [10]. The removal of faeces from horse pastures 2–3 times a week, depending on climatic conditions can be effective in reducing the acquisition of GIN infection in grazing horses [88]. However, this strategy has logistic issues in the field due to intensive requirements of labor and equipment. An alternative strategy could be a cross-grazing of horses with sheep [14, 89]. In contrast, short-term mixed grazing of horses with cattle should be limited as cattle tend to eat upper layers of the pasture, leaving the lower layers which harbor higher burdens of infective larvae per unit herbage [36]. The use of dung beetles as a biological control of strongyle nematodes in Australia remains controversial as variable results have been observed [39]. Other strategies to reduce pasture contamination may include periodic pasture destocking (i.e. spelling), separate grazing of young and old horses, and grazing with alternate hosts [36, 90]. Environmental factors appear to have a more significant influence on the occurrence of strongylids in Australian horses than the biotic factors of age, sex and breed, and so require thorough consideration while designing effective worm control strategies in horses [8].

### Anthelmintic resistance

Anthelmintic resistance (AR) of equine GINs is becoming of increasing concern in Australia. AR has been reported in equine parasites from New South Wales, Queensland and Victoria against all commonly used anthelmintic classes including benzimidazoles (cambendazole, febantel, fenbendazole, oxfendazole, oxibendazole), macrocyclic lactones (abamectin, ivermectin) and tetrahydropyrimidines (morantel, pyrantel) (see Table 3). The intensive use of a limited number of anthelmintic groups in Australia is being attributed to the emergence of anthelmintic resistant populations of equine cyathostomins and *P. equorum* [12, 18, 20–24, 79, 80, 90].

**Table 3:** Anthelmintic resistance reported in gastrointestinal nematodes of horses from Australia

Anthelmintic agent	Location	Dosage (mg/kg)^a^	%FECR (95% CL)	Parasite	Reference
Cyathostomins					
Benzimidazole	NSW, VIC	Variable	na	Cyathostomins	[90]
Cambendazole	NSW	20; oral	24	Cyathostomins	[79]
Febantel	NSW, VIC	6; oral	62	Cyathostomins	[80]
	NSW	6; oral	36	Cyathostomins	[79]
Febantel + trichlorphon	NSW, VIC	6, 40; oral	56	Cyathostomins	[80]
Fenbendazole	NSW	10; oral	Up to 0	Cyathostomins	[18]
Mebendazole	NSW, VIC	8.8; oral	43	Cyathostomins	[80]
	NSW	9; oral	40	Cyathostomins	[79]
Oxfendazole	NSW	10; oral	Up to 0 (0–na)	Cyathostomins	[12]
Oxibendazole	NSW	10; oral	Up to 9	Cyathostomins	[18]
Ivermectin	VIC	0.2; oral	na	strongyles	[22]
Morantel	NSW	9.4; oral	Up to 74 (9–na)	Cyathostomins	[12]
Dichlorvos	NSW	10; oral	Up to 0	Cyathostomins, Strongylins	[18]
*Parascaris* spp.					
Abamectin	NSW	0.2; oral	Up to -116	*Parascaris* spp.	[24]
Fenbendazole	NSW	10; oral	Up to -14 (134–na)	*Parascaris equorum*	[20]
Ivermectin	NSW	0.2; oral	57	*Parascaris* spp.	[24]
	QLD	0.2; oral	65 (47-83)	*P. equorum*	[21]
	NSW	0.2; oral	Up to 18 (-19–na)	*P. equorum*	[20]
Pyrantel	NSW	6.6; oral	Up to 26 (-49–na)	*P. equorum*	[20]

^a^Oral route

*Abbreviations*: CL, confidence limits; FECR, fecal egg count reduction; na, not available; NSW, New South Wales; QLD, Queensland; VIC, Victoria

Multiple factors have been proposed to contribute to the development of AR among equine GINs. The routine and frequent use of anthelmintics without monitoring AR is almost certainly a major contributory factor. For instance, only a small fraction (7%) of the horse owners or managers surveyed in Australia reported that they dewormed horses under their care on the basis of FEC data while the largest group (41%) reported using a blind (6–8 weeks) treatment interval practice [19]. Furthermore, about one-third of respondents dewormed their horses seasonally, biannually or annually, regardless of FECs [19]. Anthelmintic treatment at intervals equal to or shorter than ERP could select more strongly for resistant GIN populations in horses [14]. In fact, the frequent exposure of GIN populations to anthelmintic(s) may result in the elimination of susceptible parasites and refugia which otherwise ‘dilutes’ AR by providing a reservoir for drug-susceptible genes [91]. A recent survey in New South Wales revealed that horse owners perform the majority of healthcare events, including the administration of anthelmintics, without proper guidance from veterinary practitioners [6] and this might also be a factor in treatment failure and the development of AR. Inadequate dosing, heavy stocking density and poor husbandry practices (e.g. removal or faecal piles, mixing of age groups) are some of the other predisposing factors suspected to be involved in the development of AR in Australian horses [18, 90]. Rational deworming protocols based on FECs or other techniques that estimate parasite egg shedding in faeces are desperately required to effectively manage or limit of AR and for ensuring sufficient levels of refugia in horses. An investigation of management practices in Australia showed that a slow rotation of different classes of anthelmintics at intervals not less than 16 weeks could possibly help to delay the development of AR in horse GINs [90]. Furthermore, the combination of multiple anthelmintics could be effective to control nematodes with AR phenotypes [24] as multiple such products are marketed in Australia. However, combinations of multiple anthelmintics may be of limited value if the degree of resistance to individual drugs is already high [18].

### Detection of anthelmintic resistance

The lack of well-defined and reliable techniques for the detection of AR in equine GINs complicates the assessment of the status of AR in parasitic infections of horses. The faecal egg count reduction test (FECRT) is currently the most widely used method in the field for the estimation of anthelmintic efficacy/AR in horses [15, 17, 21]. This method compares pre-treatment FECs with post-treatment counts. However, geometric means which are often used when describing the results of FEC can yield biased results in contrast to arithmetic means and the use of arithmetic means has been suggested to provide a better estimate of anthelmintic efficacy [92]. Monte Carlo or bootstrap approaches have been described in an effort to address the variability in FECs [93]. These statistical techniques are, however, computationally intensive and may not be feasible when conducting FECRT in the field. Dobson et al. [94] proposed a method to determine anthelmintic efficacy that uses the total number of eggs counted rather than mean FECs (i.e. EPG values) or the number of animals in each group. This method is independent of the number of animals tested and could be valuable to estimate efficacies lower than 100% when nematode aggregation is high. For instance, counting a large number of eggs pre-treatment from high shedding animals (e.g. horses in a group with the highest FEC counts) and then egg counting from the same animals post-treatment (rather than estimating the mean) [94].

*In vitro* assays could also be useful for the detection of AR in equine GINs, although limited data are currently available. A modified egg hatch assay has been described for the detection of benzimidazole-resistant strongyle nematodes in horses [95]. This method involves the recovery of parasite eggs by sugar flotation followed by incubation with a number of anthelmintics at a range of concentrations. A recent study from Queensland has demonstrated that the 95% inhibitory concentration (IC95) is a more consistent measure of the larval response than the IC50 in the larval migration inhibition test (in a 96 well format) for the detection of ML-resistant cyathostomins [76]. A number of studies have shown promising results from *in vitro* methods used in ruminants for the estimation of anthelmintic sensitivity and AR, but further studies are required to evaluate the usefulness of these methods in horses [88, 96, 97].

### Management of AR

The frequency of anthelmintic application in horses requires a fine balance between the need to minimise faecal egg shedding and the need to curtail the development of AR in parasitic fauna. Maintaining adequate levels of refugia and finding optimum treatment strategies to reduce the number of treatments without compromising nematode control will be critical if we are to prevent an increase in parasitic disease in horses. Regular treatment of horses in New South Wales every 8–10 weeks has been shown to delay the re-establishment of adult (egg-laying) nematodes, compared with summer treatment regimens [75]. However, the optimum treatment interval may vary depending on a range of factors including the type of pasture, grazing practices, stocking density, time of the year, and climatic conditions [17]. Limiting the exposure of horses to parasitic larvae on the pasture could further aid in minimising the number of treatments required. Based on simulated Australian conditions, a rapid rotation between a triple-combination product (benzimidazole + levamisole + abamectin) and monepantel has been proposed for sheep [94]. However, further studies are required to tailor-design treatment guidelines for Australian horse owners/managers for effective control of GINs as well as minimise AR.

To delay the development of AR, a selective (targeted) anthelmintic treatment (SAT) has been proposed as an alternative deworming approach to control cyathostomin [14, 16, 98]. SAT involves treating only those horses which carry moderate to high FECs (high shedders), leaving a proportion of the worm population in refugia in untreated horses [16]. The SAT approach accounts for the over dispersed distribution of GINs within the horse population and through the diluting effects of refugia, slows the development of AR [14, 99, 100]. Intensive studies are required to validate SAT approaches in horse enterprises under local climatic conditions in Australia.

## Conclusions

GIN infections remain a major health concern in horses worldwide. About 45 nematode species have been reported in horses from various regions of Australia, of which approximately two-thirds belong to cyathostomins (Table 1). Although, GINs infecting Australian horses have been the subject of occasional studies over the past few decades, the effective control of GIN infections is hampered by a generalised lack of knowledge in various disciplines of equine parasitology, including (i) the current nation-wide prevalence and abundance status of equine GINs; (ii) the effect of biotic/abiotic epidemiological factors (e.g. breed, age, season, husbandry practices etc.) on GIN prevalence in horses; (iii) economic losses associated with clinical and subclinical GIN infections in horses; and (iv) the current status of AR in nematode populations infecting horses. Additionally, there is currently a lack of the following key elements: (i) standard guidelines on the use of anthelmintics in stabled and paddocked horses across various climatic zones; and (ii) recommendations (tailored for use under local agroclimatic conditions) on best worm control practices for GIN management in horses. Therefore, coordinated and focused research is required to fill our knowledge gaps in these areas to maximise equine health and minimise economic losses associated with the parasitic infections in Australia. GINs remain a significant health, welfare and performance concern in Australian horses due to our limited local knowledge about these parasites. Very limited information is available on the current epidemiology of equine GINs, the efficacy of anthelmintic drugs, suitable treatment intervals based on ERP under various climatic conditions, and the status of AR. Although worm control practices based on the knowledge from other regions of the world have shown some degree of success to control equine GINs in Australia, treatment failure and AR continue to rise in the country [12, 18, 20–22, 24, 79, 80, 90]. Given the lack of any new anthelmintic classes for the equine industry on the horizon, it is necessary to thoroughly review horse management practices in Australia with the aim of curtailing the development of resistance to all anthelmintic classes. Thus, the development of Australia-specific control and treatment strategies based on local epidemiological data are of utmost importance for effective management of GIN infections in horses. The use of rational treatment strategies such as SAT approach, integrated with non-chemical management practices could play a crucial role in achieving sustainable control of GIN infections in horses. Future studies should be directed toward investigating the epidemiology and clinicopathology of equine GINs as well as worm control strategies and anthelmintic treatment protocols for effective management of these parasites in Australia.

## Additional files

**Additional file 1: Table S1.** PRISMA 2009 checklist.

**Additional file 2: Table S2.** Studies on parasite(s) identified from horses in Australia.

## Abbreviations

AR: anthelmintic resistance
EPG: eggs per gram of faeces
FEC: faecal egg count
FECRT: faecal egg count reduction test
GIN: gastrointestinal nematode
ML: macrocyclic lactone
PCR: polymerase chain reaction
RFLP: fragment length polymorphism
SAT: selective anthelmintic treatment
SSCP: single-strand conformation polymorphism
