# Supplementary material for: Systematic review of gastrointestinal nematodes of horses from Australia
Source: Parasit Vectors. 2019 Apr 29;12:188. doi: 10.1186/s13071-019-3445-4 (PMC6489199; doi:10.1186/s13071-019-3445-4)
Supplement: Supplementary file 2 — Additional file 2: Table S2. Studies on parasite(s) identified from horses in Australia. [file 13071_2019_3445_MOESM2_ESM.docx]

**Additional file 2: Table S2.** Gastrointestinal nematodes identified from horses in Australia

| **Parasite type/spp. (former name)** | **Location** | **Horse type(s)** | **Age in years (age group)** | **Proportion** | **Percent prevalence** | **Method of detection** | **EPG or parasites per host range (mean / median)** | **Reference(s)** | **Museum collection identification #** |
| --- | --- | --- | --- | --- | --- | --- | --- | --- | --- |
| **Cyathostomins** | | | | | | | | | |
| *Coronocyclus coronatus*  (*Cyathostomum coronatum*) | WA | B, P, Ph, Sb, Tb | 1-23 (C, G, M) | 10/29 | 34 | PM | 88-6691 (1045) | (Boxell *et al.*, 2004) | SAM AHC 24536, 31554, QM GL 10719, USNPC 88376 |
|  | VIC | H, Tb | ≤ 2 - ≥ 15 | +/150 | 43 | PM | (1720) | (Bucknell *et al.*, 1995) |  |
|  | QLD | NA | 0 - ˃ 15 | +/57 | 65 | PM | (480) | (Mfitilodze & Hutchinson, 1990; Mfitilodze & Hutchinson, 1985) |  |
| *Co. labiatus* (*Cyathostomum labiatum* | WA | B, P, Ph, Sb, Tb | 1-23 (C, G, M) | 2/29 | 7 | PM | 100-468 (216) | (Boxell *et al.*, 2004) | SAM AHC 24520, 31555, QM GL 10721, USNPC 88377 |
|  | VIC | NA | NA | + | NA | PCR | NA | (Gasser *et al.*, 1996) |  |
|  | VIC | H, Tb | ≤ 2 - ≥ 15 | +/150 | 13 | PM | (5170) | (Bucknell *et al.*, 1995) |  |
|  | QLD | NA | 0 - ˃ 15 | +/57 | 30 | PM | (620) | (Mfitilodze & Hutchinson, 1990; Mfitilodze & Hutchinson, 1985) |  |
| *Co. labratus* (*Cy. labratum*) | WA | B, P, Ph, Sb, Tb | 1-23 (C, G, M) | 1/29 | 4 | PM | 105 | (Boxell *et al.*, 2004) | SAM AHC 15241, 24530, 31556 |
|  | VIC | H, Tb | ≤ 2 - ≥ 15 | +/150 | 13 | PM | (4270) | (Bucknell *et al.*, 1995) |  |
|  | QLD | NA | 0 - ˃ 15 | +/57 | NA | 2 | (20) | (Mfitilodze & Hutchinson, 1990; Mfitilodze & Hutchinson, 1985) |  |
|  | VIC | NA | NA | + | NA | PCR | NA | (Gasser *et al.*, 1996) |  |
| Cyathostomes | QLD | NA | 0 - ˃ 15 | +/NA | NA | MM | NA | (Mfitilodze & Hutchinson, 1988) |  |
|  | WA | H, P, Sb, Tb | 0.5-15 | 68/138 | 49 | PM | NA | (Dunsmore & Sue, 1985) |  |
| *Cyathostomum catinatum* | WA | B, P, Ph, Sb, Tb | 1-23 (C, G, M) | 22/29 | 76 | PM | 65-58435 (3665) | (Boxell *et al.*, 2004) | SAM AHC 15242, 24527, QM G 10718, 214929, USNPC 88374 |
|  | VIC | NA | NA | + | NA | PCR | NA | (Gasser *et al.*, 1996; Hung *et al.*, 1999b) |  |
|  | VIC | H, Tb | ≤ 2 - ≥ 15 | +/150 | 68 | PM | (15700) | (Bucknell *et al.*, 1995) |  |
|  | QLD | NA | 0 - ˃ 15 | +/57 | 76 | PM | (4080) | (Mfitilodze & Hutchinson, 1990; Mfitilodze & Hutchinson, 1985) |  |
| *Cy. pateratum* | VIC | H, Tb | ≤ 2 - ≥ 15 | +/150 | 16 | PM | (4840) | (Bucknell *et al.*, 1995) | SAM AHC 24526, 31558, QM G 214930, GL 10720, USNPC 88375 |
|  | QLD | NA | 0 - ˃ 15 | +/57 | 33 | PM | (1540) | (Mfitilodze & Hutchinson, 1990; Mfitilodze & Hutchinson, 1985) |  |
| *Cyathostomum spp.* | VIC | P | (G, M) | +/18 | NA | LC | ≥ 1000 | (Flanagan *et al.*, 2013) |  |
| *Cylicocyclus ashworthi* | VIC | NA | NA | NA | NA | PCR | NA | (Hung *et al.*, 1997) | SAM AHC 31599 |
| *Cc. auriculatus* | VIC | H, Tb | ≤ 2 - ≥ 15 | +/150 | 1 | PM | (100) | (Bucknell *et al.*, 1995) | SAM AHC 24517 |
| *Cc. brevicapsulatus* | VIC | H, Tb | ≤ 2 - ≥ 15 | +/150 | 26 | PM | (27300) | (Bucknell *et al.*, 1995) | SAM AHC 15249, 24516, QM GL 10710, USNPC 88368 |
|  | QLD | NA | 0 - ˃ 15 | +/57 | 22 | PM | (230) | (Mfitilodze & Hutchinson, 1990; Mfitilodze & Hutchinson, 1985) |  |
| *Cc. elongatus* | VIC | H, Tb | ≤ 2 - ≥ 15 | +/150 | 4 | PM | (2090) | (Bucknell *et al.*, 1995) | SAM AHC 15248, 24523, QM GL 10712, USNPC 88369 |
|  | QLD | NA | 0 - ˃ 15 | +/57 | 9 | PM | (900) | (Mfitilodze & Hutchinson, 1990; Mfitilodze & Hutchinson, 1985) |  |
| *Cc. insignis* (incl. *Cylicocyclus adersi*)* | WA | B, P, Ph, Sb, Tb | 1-23 (C, G, M) | 3/29 | 10 | PM | 800-29662 (6864) | (Boxell *et al.*, 2004) | SAM AHC 15246, 24513, 31560, QM GL 10713, UNSPC 88370 |
|  | VIC | NA | NA | + | NA | PCR | NA | (Gasser *et al.*, 1996) |  |
|  | VIC | H, Tb | ≤ 2 - ≥ 15 | +/150 | 17 | PM | (4900) | (Bucknell *et al.*, 1995) |  |
|  | QLD | NA | 0 - ˃ 15 | +/57 | 41 | PM | (1580) | (Mfitilodze & Hutchinson, 1990; Mfitilodze & Hutchinson, 1985) |  |
| *Cc. leptostomus* | VIC | NA | NA | + | NA | PCR | NA | (Gasser *et al.*, 1996) | SAM AHC 24521, 31561, QM GL 10714, USNPC 88371 |
|  | VIC | H, Tb | ≤ 2 - ≥ 15 | +/150 | 41 | PM | (12600) | (Bucknell *et al.*, 1995) |  |
|  | QLD | NA | 0 - ˃ 15 | +/57 | 41 | PM | (1460) | (Mfitilodze & Hutchinson, 1990; Mfitilodze & Hutchinson, 1985) |  |
| *Cc. nassatus* | WA | B, P, Ph, Sb, Tb | 1-23 (C, G, M) | 21/29 | 72 | PM | 80-257460 (5450) | (Boxell *et al.*, 2004)* | SAM AHC 15247, 24514, 31562, QM GL 10715, USNPC 88372 |
|  | VIC | NA | NA | + | NA | PCR | NA | (Gasser *et al.*, 1996; Hung *et al.*, 1997; Hung *et al.*, 1999b) |  |
|  | VIC | H, Tb | ≤ 2 - ≥ 15 | +/150 | 54 | PM | (10270) | (Bucknell *et al.*, 1995) |  |
|  | QLD | NA | 0 - ˃ 15 | +/57 | 67 | PM | (5380) | (Mfitilodze & Hutchinson, 1990; Mfitilodze & Hutchinson, 1985) |  |
| *Cc. radiatus* | VIC | H, Tb | ≤ 2 - ≥ 15 | +/150 | 4 | PM | (2780) | (Bucknell *et al.*, 1995) | SAM AHC 24522, 31563, QM GL 10716, USNPC 88373 |
|  | QLD | NA | 0 - ˃ 15 | +/57 | 33 | PM | (2250) | (Mfitilodze & Hutchinson, 1990; Mfitilodze & Hutchinson, 1985) |  |
| *Cc. ultrajectinus* | WA | B, P, Ph, Sb, Tb | 1-23 (C, G, M) | 1/29 | 4 | PM | 209 | (Boxell *et al.*, 2004) | QM G 214927, GL 10717 |
|  | VIC | H, Tb | ≤ 2 - ≥ 15 | +/150 | 1 | PM | (80) | (Bucknell *et al.*, 1995) |  |
|  | QLD | NA | NA | +/57 | 9 | PM | (850) | (Mfitilodze & Hutchinson, 1990; Mfitilodze & Hutchinson, 1985) |  |
| *Cylicodontophorus bicoronatus* | VIC | H, Tb | ≤ 2 - ≥ 15 | +/150 | 3 | PM | (1300) | (Bucknell *et al.*, 1995) | SAM AHC 24539, QM G 214926 |
|  | QLD | NA | NA | +/57 | 4 | PM | (60) | (Mfitilodze & Hutchinson, 1990; Mfitilodze & Hutchinson, 1985) |  |
| *Cylicostephanus asymetricus* | QLD | NA | NA | +/57 | 2 | PM | 60 | (Mfitilodze & Hutchinson, 1990; Mfitilodze & Hutchinson, 1985) | QM G 214936, GL 10727 |
| *Cs. calicatus* | WA | B, P, Ph, Sb, Tb | 1-23 (C, G, M) | 9/29 | 31 | PM | 264-10655 (848) | (Boxell *et al.*, 2004) | SAM AHC 24515, 31565, QM GL 10723, USNPC 88378 |
|  | VIC | NA | NA | + | NA | PCR | NA | (Gasser *et al.*, 1996) |  |
|  | VIC | H, Tb | ≤ 2 - ≥ 15 | +/150 | 48 | PM | (2730) | (Bucknell *et al.*, 1995) |  |
|  | QLD | NA | NA | +/57 | 70 | PM | (860) | (Mfitilodze & Hutchinson, 1990; Mfitilodze & Hutchinson, 1985) |  |
| *Cs. goldi* | WA | B, P, Ph, Sb, Tb | 1-23 (C, G, M) | 14/29 | 48 | PM | 33-150007 (1564) | (Boxell *et al.*, 2004) | SAM AHC 15230, 24519, 31566, QM GL 10724, USNPC 88379 |
|  | VIC | NA | NA | + | NA | PCR | NA | (Gasser *et al.*, 1996; Hung *et al.*, 1999a) |  |
|  | VIC | H, Tb | ≤ 2 - ≥ 15 | +/150 | 51 | PM | (6360) | (Bucknell *et al.*, 1995) |  |
|  | QLD | NA | NA | +/57 | 43 | PM | (1200) | (Mfitilodze & Hutchinson, 1990; Mfitilodze & Hutchinson, 1985) |  |
| *Cs. hybridus* | QLD | NA | 0 - ˃ 15 | +/57 | 4 | PM | (360) | (Mfitilodze & Hutchinson, 1990; Mfitilodze & Hutchinson, 1985) |  |
| *Cs. longibursatus* | WA | B, P, Ph, Sb, Tb | 1-23 (C, G, M) | 17/29 | 59 | PM | 35-18470 (2829) | (Boxell *et al.*, 2004) | SAM AHC 24537, 31567, QM GL 10722, USNPC 88380 |
|  | VIC | NA | NA | + | NA | PCR | NA | (Gasser *et al.*, 1996; Hung *et al.*, 1999a) |  |
|  | VIC | H, Tb | ≤ 2 - ≥ 15 | +/150 | 76 | PM | (23600) | (Bucknell *et al.*, 1995) |  |
|  | QLD | NA | 0 - ˃ 15 | +/57 | 67 | PM | (4580) | (Mfitilodze & Hutchinson, 1990; Mfitilodze & Hutchinson, 1985) |  |
| *Cs. minutus* | WA | B, P, Ph, Sb, Tb | 1-23 (C, G, M) | 3/29 | 10 | PM | 105-10334 (983) | (Boxell *et al.*, 2004) | SAM AHC 15232, 24518, 31568, QM GL 10725, USNPC 88381 |
|  | VIC | H, Tb | ≤ 2 - ≥ 15 | +/150 | 36 | PM | (11400) | (Bucknell *et al.*, 1995) |  |
|  | QLD | NA | 0 - ˃ 15 | +/57 | 26 | PM | (1310) | (Mfitilodze & Hutchinson, 1990; Mfitilodze & Hutchinson, 1985) |  |
| *Gyalocephalus capitatus* | VIC | H, Tb | ≤ 2 - ≥ 15 | +/150 | 1 | PM | (750) | (Bucknell *et al.*, 1995) | SAM AHC 6180, USNPC 88386 |
|  | QLD | NA | 0 - ˃ 15 | +/57 | 11 | PM | (60) | (Mfitilodze & Hutchinson, 1990; Mfitilodze & Hutchinson, 1985) |  |
| *Parapoteriostomum euproctus* (*Cylicodontophorus euproctus*) | VIC | H, Tb | ≤ 2 - ≥ 15 | +/150 | 1 | PM | (400) | (Bucknell *et al.*, 1995) | SAM AHC 31569, QM GL 10728, USNPC 88385 |
|  | QLD | NA | NA | +/57 | 15 | PM | (850) | (Mfitilodze & Hutchinson, 1990; Mfitilodze & Hutchinson, 1985) |  |
| *Pp. mettami* (*Cylicodontophorus mettami*) | VIC | H, Tb | ≤ 2 - ≥ 15 | +/150 | 3 | PM | (1330) | (Bucknell *et al.*, 1995) | SAM AHC 24531, 31570, QM GL 10729 |
|  | QLD | NA | NA | +/57 | 4 | PM | (20) | (Mfitilodze & Hutchinson, 1990; Mfitilodze & Hutchinson, 1985) |  |
| *Petrovinema poculatum* (*Cylicostephanus poculatus*) | VIC | H, Tb | ≤ 2 - ≥ 15 | +/150 | 2 | PM | (1110) | (Bucknell *et al.*, 1995) | SAM AHC 24524, QM GL 10726, USNPC 88382 |
|  | QLD | NA | NA | +/57 | 9 | PM | (30) | (Mfitilodze & Hutchinson, 1990; Mfitilodze & Hutchinson, 1985) |  |
| *Poteriostomum imparidentatum* | VIC | NA | 0 - ˃ 15 | + | NA | PCR | NA | (Gasser *et al.*, 1996) | SAM AHC 24538, 31571, QM GL 10731, USNPC 88383 |
|  | VIC | H, Tb | ≤ 2 - ≥ 15 | +/150 | 3 | PM | (860) | (Bucknell *et al.*, 1995) |  |
|  | QLD | NA | NA | +/57 | 24 | PM | (810) | (Mfitilodze & Hutchinson, 1990; Mfitilodze & Hutchinson, 1985) |  |
| *P. ratzii* | VIC | NA | NA | + | NA | PCR | NA | (Gasser *et al.*, 1996) | SAM AHC 31572, QM GL 10732, USNPC 88384 |
|  | VIC | H, Tb | ≤ 2 - ≥ 15 | +/150 | 1 | PM | NA | (Bucknell *et al.*, 1995) |  |
|  | QLD | NA | 0 - ˃ 15 | +/57 | 11 | PM | (100) | (Mfitilodze & Hutchinson, 1990; Mfitilodze & Hutchinson, 1985) |  |
| *P. skrjabini* | QLD | NA | 0 - ˃ 15 | +/57 | 2 | PM | (20) | (Mfitilodze & Hutchinson, 1990; Mfitilodze & Hutchinson, 1985) |  |
| Strongyles | VIC | P | (G, M) | 55/106 | 52 | PM | 0-3750 (422) | (Flanagan *et al.*, 2013) |  |
|  | QLD | NA | NA | + | NA | MM | Up to 1400 | (Lavers, 1964) |  |
| **Strongylins** | | | | | | | | | |
| *Craterostomum acuticaudatum* | VIC | H, Tb | ≤ 2 - ≥ 15 | +/150 | 7 | PM | (1580) | (Bucknell *et al.*, 1995) | SAM AHC 31557, QM G 214937 |
| *Oesophagodontus robustus* | VIC | NA | NA | + | NA | PCR | NA | (Gasser *et al.*, 1996) | QM G 214939, GL 10746 |
|  | QLD | NA | NA | +/57 | 2 | PM | (20) | (Mfitilodze & Hutchinson, 1990; Mfitilodze & Hutchinson, 1985) |  |
| *Strongylus edentatus* | VIC | NA | NA | + | NA | PCR | NA | (Gasser *et al.*, 1996) | SAM AHC 2444, 15235, 15382, 24535, USNPC 99390 |
|  | VIC | H, Tb | ≤ 2 - ≥ 15 | +/150 | 23 | PM | (1150) | (Bucknell *et al.*, 1995) |  |
|  | WA | H, P, Sb, Tb | 0.5-15 | 35/138 | 26 | PM | 0-55 | (Dunsmore & Sue, 1985) |  |
|  | QLD | NA | 0 - ˃ 15 | +/57 | 22 | PM | (70) | (Mfitilodze & Hutchinson, 1990; Mfitilodze & Hutchinson, 1985) |  |
|  | QLD | NA | 0 - ˃ 10 | +/138 | 18 | PM | 1-31 | (English, 1979) |  |
| *Strongylus equinus* | VIC | NA | NA | + | NA | PCR | NA | (Gasser *et al.*, 1996) | SAM AHC 24529, USNPC 88391 |
|  | VIC | H, Tb | ≤ 2 - ≥ 15 | +/150 | 3 | PM | NA | (Bucknell *et al.*, 1995) |  |
|  | QLD | NA | NA | +/57 | 22 | PM | (330) | (Mfitilodze & Hutchinson, 1990; Mfitilodze & Hutchinson, 1985) |  |
|  | QLD | NA | 0 - ˃ 10 | +/138 | 70 | PM | 1-172 | (English, 1979) |  |
| *Strongylus vulgaris* | VIC | NA | NA | + | NA | PCR | NA | (Gasser *et al.*, 1996; Hung *et al.*, 1999b) | SAM AHC 24532, USNPC 88392 |
|  | VIC | H, Tb | ≤ 2 - ≥ 15 | +/150 | 23 | PM | (410) | (Bucknell *et al.*, 1995) |  |
|  | WA | H, P, Sb, Tb | 0.5-15 | 31/138 | 22 | PM | 0-235 | (Dunsmore & Sue, 1985) |  |
|  | QLD | NA | 0 - ˃ 15 | +/57 | 28 | PM | (360) | (Mfitilodze & Hutchinson, 1990; Mfitilodze & Hutchinson, 1985) |  |
|  | QLD | NA | 0 - ˃ 10 | +/138 | 88 | PM | 1-629 | (English, 1979) |  |
|  | VIC | NA | NA | +/147 | NA | NA | NA | (Arundel, 1978) |  |
| *Triodontophorus brevicauda* | VIC | H, Tb | ≤ 2 - ≥ 15 | +/150 | 3 | PM | (660) | (Bucknell *et al.*, 1995) | SAM AHC 15244, 24512, QM GL 10739 |
|  | QLD | NA | 0 - ˃ 15 | +/57 | 11 | PM | (460) | (Mfitilodze & Hutchinson, 1990; Mfitilodze & Hutchinson, 1985) |  |
| *T. minor* | QLD | NA | 0 - ˃ 15 | +/57 | 15 | PM | (210) | (Mfitilodze & Hutchinson, 1990; Mfitilodze & Hutchinson, 1985) | USNPC 88387, QM G 214942, GL 10738 |
| *T. nipponicus* | QLD | NA | 0 - ˃ 15 | +/57 | 13 | PM | (390) | (Mfitilodze & Hutchinson, 1990; Mfitilodze & Hutchinson, 1985) | SAM AHC 15245, QM GL 10740, USNPC 88388 |
| *T. serratus* | WA | B, P, Ph, Sb, Tb | 1-23 (C, G, M) | 3/29 | 10 | PM | 60-9310 (380) | (Boxell *et al.*, 2004) | SAM AHC 24533, QM G 214943, GL 10737, USNPC 88389 |
|  | VIC | NA | NA | + | NA | PCR | NA | (Gasser *et al.*, 1996) |  |
|  | VIC | H, Tb | ≤ 2 - ≥ 15 | +/150 | 8 | PM | (450) | (Bucknell *et al.*, 1995) |  |
|  | QLD | NA | 0 - ˃ 15 | +/57 | 30 | PM | (570) | (Mfitilodze & Hutchinson, 1990; Mfitilodze & Hutchinson, 1985) |  |
| *T. tenuicollis* | VIC | H, Tb | ≤ 2 - ≥ 15 | +/150 | 4 | PM | (200) | (Bucknell *et al.*, 1995) | SAM AHC 24528, QM G 214941 |
| *Triodontophorus* spp. | QLD | NA | 0 - ˃ 10 | +/138 | 23 | PM | 1-125 | (English, 1979) |  |
| **Non-strongylids** | | | | | | | | | |
| *Draschia megastoma* | VIC | H, Tb | ≤ 2 - ≥ 15 | +/150 | 5 | PM | (170) | (Bucknell *et al.*, 1995) | SAM AHC 15237, 15240, QM GL 10742 |
|  | QLD | NA | 0 - ˃ 15 | +/57 | 39 | PM | (960) | (Mfitilodze & Hutchinson, 1989) |  |
|  | WA | H, P, Sb, Tb | 0.5-15 | 92/140 | 66 | PM | NA | (Dunsmore & Sue, 1985) |  |
|  | QLD | NA | NA | 115/280 | 41 | PM | NA | (Waddell, 1969) |  |
|  | NSW, SA | NA | NA | + | NA | PM | NA | (Seddon, 1950) |  |
| *Habronema muscae* | WA | B, P, Ph, Sb, Tb | 1-23 (C, G, M) | 13/29 | 45 | PM | 4-800 (122) | (Boxell *et al.*, 2004) | SAM AHC 15236, 24525, QM GL 10741, USNPC 88422 |
|  | VIC | H, Tb | ≤ 2 - ≥ 15 | +/150 | 13 | PM | 280 | (Bucknell *et al.*, 1995) |  |
|  | QLD | NA | 0 - ˃ 15 | +/57 | 43 | PM | (500) | (Mfitilodze & Hutchinson, 1989) |  |
|  | WA | H, P, Sb, Tb | 0.5-15 | +/140 | 35 | PM | NA | (Dunsmore & Sue, 1985) |  |
|  | QLD | NA | NA | 202/280 | 72 | PM | NA | (Waddell, 1969) |  |
| *H. majus* (*H. microstoma*) | VIC | H, Tb | ≤ 2 - ≥ 15 | +/150 | 2 | PM | 1860 | (Bucknell *et al.*, 1995) | SAM AHC 21024 |
| *Trichostrongylus axei* | VIC | H, Tb | ≤ 2 - ≥ 15 | +/150 | 51 | PM | (8540) | (Bucknell *et al.*, 1995) |  |
| *Oxyuris equi* | WA | B, P, Ph, Sb, Tb | 1-23 (C, G, M) | 2/29 | 7 | PM | 1425-10977 (3959) | (Boxell *et al.*, 2004) | SAM AHC 24534, USNPC 57173, 88421 |
|  | VIC | H, Tb | ≤ 2 - ≥ 15 | +/150 | 7 | PM | (7600) | (Bucknell *et al.*, 1995) |  |
|  | QLD | NA | 0 - ˃ 15 | +/57 | 26 | PM | (1310) | (Mfitilodze & Hutchinson, 1989) |  |
|  | WA | H, P, Sb, Tb | 0.5-15 | 1/NA | NA | PM | NA | (Dunsmore & Sue, 1985) |  |
| *Parascaris equorum* | NSW | Sb, Tb | (F) | 147/252 | 58 | MM | 0-≥100 | (Armstrong *et al.*, 2014) | QM GL 5012-5026, 10745 |
|  | WA | B, P, Ph, Sb, Tb | 1-23 (C, G, M) | 4/29 | 14 | PM | 9-40 (23) | (Boxell *et al.*, 2004) |  |
|  | VIC | H, Tb | NA | +/150 | 5 | PM | (14) | (Bucknell *et al.*, 1995) |  |
|  | QLD | NA | 0 - ˃ 15 | +/57 | 15 | PM | (20) | (Mfitilodze & Hutchinson, 1989) |  |
|  | WA | H, P, Sb, Tb | 0.5-4 | 14/142 | 10 | PM | 0-48 (12) | (Dunsmore & Sue, 1985) |  |
| *Strongyloides westeri* | QLD | NA | 0 - ˃ 15 | +/57 | 6 | PM | NA | (Mfitilodze & Hutchinson, 1989) |  |
|  | VIC | NA | NA | + | NA | NA | NA | (Arundel, 1978) |  |
| *Probstmayria vivipara* | QLD | NA | 0 - ˃ 15 | +/57 | 2 | PM | NA | (Mfitilodze & Hutchinson, 1989) |  |

Abbreviations: B, broodmares; C, colts; EPG, eggs per gram faeces; F, foals; H, hacks; G, gelding; LC, larval culture; M, mare; MM, Macmaster; NSW, New South Wales; P, pony; Ph; pet/performance horses; PM, post-mortem examination and adult worm/larval identification; QM, Queensland Museum; S, stallion; SA, South Australia; SAM, South Australian Museum; Sb, standardbred; Tb, thoroughbred; USNPC, United States National Parasite Collection; VIC, Victoria; WA, Western Australia .

*Specimens reported by Mfitilodze & Hutchinson (1989) as *Cc. adersi* were re-identified as *Cc. insignis* by R. Lichtenfels.

**Rahman (1976) reported *Cyathostomum tetracanthum*, *Cylicocyclus gyalocephaloides* (a parasite of zebras) and *Cylicocyclus triramosus* (also a parasite of zebras) from Australian horses. His specimens in QM (G214915, G214290, G214921) are too poorly preserved to permit identification.

**References**

**Armstrong, S. K., Woodgate, R. G., Gough, S., Heller, J., Sangster, N. C. and Hughes, K. J.** (2014). The efficacy of ivermectin, pyrantel and fenbendazole against *Parascaris equorum* infection in foals on farms in Australia. *Veterinary Parasitology,* **205**, 575-580. doi: 10.1016/j.vetpar.2014.08.028.

**Arundel, J.** (1978). *Parasitic diseases of the horse,* Post-Graduate Committee in Veterinary Science, PO Box A561, Sydney, Australia.

**Boxell, A. C., Gibson, K. T., Hobbs, R. P. and Thompson, R. C. A.** (2004). Occurrence of gastrointestinal parasites in horses in metropolitan Perth, Western Australia. *Australian Veterinary Journal,* **82**, 91-95. doi: 10.1111/j.1751-0813.2004.tb14653.x.

**Bucknell, D., Gasser, R. and Beveridge, I.** (1995). The prevalence and epidemiology of gastrointestinal parasites of horses in Victoria, Australia. *International Journal for Parasitology,* **25**, 711-724.

**Dunsmore, J. and Sue, L. P. J.** (1985). Prevalence and epidemiology of the major gastrointestinal parasites of horses in Perth, Western Australia. *Equine Veterinary Journal,* **17**, 208-213.

**English, A. W.** (1979). The epidemiology of equine strongylosis in southern Queensland. 3. Seasonal variation in arterial populations of *Strongylus vulgaris*, and the prevalence of some helminths. *Aust Vet J,* **55**, 310-314.

**Flanagan, K., Morton, J. and Sandeman, R.** (2013). Prevalence of infestation with gastrointestinal nematodes in Pony Club horses in Victoria. *Australian Veterinary Journal,* **91**, 241-245.

**Gasser, R. B., Stevenson, L. A., Chilton, N. B., Nansen, P., Bucknell, D. G. and Beveridge, I.** (1996). Species markers for equine strongyles detected in intergenic rDNA by PCR-RFLP. *Mol Cell Probes,* **10**, 371-378. doi: 10.1006/mcpr.1996.0050.

**Hung, G.-C., Chilton, N., Beveridge, I., McDonnell, A., Lichtenfels, J. and Gasser, R.** (1997). Molecular delineation of *Cylicocyclus nassatus* and *C. ashworthi* (Nematoda: Strongylidae). *International Journal for Parasitology,* **27**, 601-605.

**Hung, G.-C., Chilton, N. B., Beveridge, I. and Gasser, R. B.** (1999a). Secondary structure model for the ITS-2 precursor rRNA of strongyloid nematodes of equids: implications for phylogenetic inference. *International Journal for Parasitology,* **29**, 1949-1964.

**Hung, G. C., Gasser, R. B., Beveridge, I. and Chilton, N. B.** (1999b). Species-specific amplification by PCR of ribosomal DNA from some equine strongyles. *Parasitology,* **119**, 69-80. doi: 10.1017/s0031182099004497.

**Lavers, D.** (1964). The problem of internal parasites in working horses. *Australian Veterinary Journal,* **40**, 150-156.

**Mfitilodze, M. and Hutchinson, G.** (1989). Prevalence and intensity of non‐strongyle intestinal parasites of horses in northern Queensland. *Australian Veterinary Journal,* **66**, 23-26.

**Mfitilodze, M. and Hutchinson, G.** (1990). Prevalence and abundance of equine strongyles (Nematoda: Strongyloidea) in tropical Australia. *The Journal of parasitology*, 487-494.

**Mfitilodze, M. W. and Hutchinson, G. W.** (1985). The site distribution of adult strongyle parasites in the large intestines of horses in tropical Australia. *Int J Parasitol,* **15**, 313-319.

**Mfitilodze, M. W. and Hutchinson, G. W.** (1988). Development of free-living stages of equine strongyles in faeces on pasture in a tropical environment. *Vet Parasitol,* **26**, 285-296.

**Seddon, H.** (1950). *Diseases Of Domestic Animals In Australia Pt. 1,* AH Pettifer, Sydney, Australia.

**Waddell, A.** (1969). A survey of *Habronema* spp and the identification of third‐stage larvae of *Habronema megastoma* and *Habronema muscae* in section. *Australian Veterinary Journal,* **45**, 20-21.
